# Association of Maternal Factors and HIV Infection With Innate Cytokine Responses of Delivering Mothers and Newborns in Mozambique

**DOI:** 10.3389/fmicb.2020.01452

**Published:** 2020-07-14

**Authors:** Gemma Moncunill, Carlota Dobaño, Raquel González, Kinga K. Smolen, Maria N. Manaca, Reyes Balcells, Chenjerai Jairoce, Pau Cisteró, Anifa Vala, Esperança Sevene, María Rupérez, John J. Aponte, Eusébio Macete, Clara Menéndez, Tobias R. Kollmann, Alfredo Mayor

**Affiliations:** ^1^ISGlobal, Hospital Clínic – Universitat de Barcelona, Barcelona, Spain; ^2^Centro de Investigação em Saúde de Manhiça (CISM), Maputo, Mozambique; ^3^Department of Pediatrics, BC Children’s Hospital, The University of British Columbia, Vancouver, BC, Canada; ^4^Department of Experimental Medicine, The University of British Columbia, Vancouver, BC, Canada; ^5^Consorcio de Investigación Biomédica en Red de Epidemiología y Salud Pública (CIBERESP), Instituto de Salud Carlos III, Madrid, Spain; ^6^Department of Physiological Science, Clinical Pharmacology, Faculty of Medicine, Eduardo Mondlane University, Maputo, Mozambique

**Keywords:** cytokines, pattern recognition receptors, innate immunity, cord, pregnant women, anemia, HIV, HIV exposed uninfected

## Abstract

Maternal factors and exposure to pathogens have an impact on infant health. For instance, HIV exposed but uninfected infants have higher morbidity and mortality than HIV unexposed infants. Innate responses are the first line of defense and orchestrate the subsequent adaptive immune response and are especially relevant in newborns. To determine the association of maternal HIV infection with maternal and newborn innate immunity we analyzed the cytokine responses upon pattern recognition receptor (PRR) stimulations in the triad of maternal peripheral and placental blood as well as in cord blood in a cohort of mother-infant pairs from southern Mozambique. A total of 48 women (35 HIV-uninfected and 13 HIV-infected) were included. Women and infant innate responses positively correlated with each other. Age, gravidity and sex of the fetus had some associations with spontaneous production of cytokines in the maternal peripheral blood. HIV-infected women not receiving antiretroviral therapy (ART) before pregnancy showed decreased IL-8 and IL-6 PRR responses in peripheral blood compared to those HIV-uninfected, and PRR hyporesponsiveness for IL-8 was also found in the corresponding infant’s cord blood. HIV infection had a greater impact on placental blood responses, with significantly increased pro-inflammatory, T_*H*_1 and T_*H*_17 PRR responses in HIV-infected women not receiving ART before pregnancy compared to HIV-uninfected women. In conclusion, innate response of the mother and her newborn was altered by HIV infection in the women who did not receive ART before pregnancy. As these responses could be related to birth outcomes, targeted innate immune modulation could improve maternal and newborn health.

## Introduction

Globally, there were 2.5 million estimated deaths in children within the first month of life in 2017 (WHO, 2017), mostly in low-income countries. Infectious diseases are one of the main causes of mortality in children under the age of 5 (WHO, 2017). It is well known that newborns and infants are more susceptible to infections and severity of infectious diseases than adults (Brook et al., 2017). Infants’ vulnerability reflects differences in the immune system in early life compared to adults, which may result in a slower immune response defending against infecting pathogens yet increased immunopathology upon infection. The underlying specific causes are yet to be elucidated (Kollmann et al., 2017).

The immune system of newborns and infants is adapted to the peculiarities of this age period. Newborns come from a tolerogenic fetal environment and suddenly they are exposed to the maternal and environmental microbiome, which requires extensive efforts to maintain homeostasis. Studies of immune ontogeny have demonstrated that immune development undergoes intense changes during the early period of life, yet follows a stable developmental trajectory (Olin et al., 2018; Lee et al., 2019). The changes during this developmental phase, which are key for health and disease, are probably driven by a range of environmental exposures. Maternal factors and exposure to pathogens during pregnancy have a clear impact on the health outcomes of infants. A clear example is malaria during pregnancy. Infants exposed to *Plasmodium in utero* have an increased risk for malaria (Schwarz et al., 2008) as well as non-malaria infections (Rachas et al., 2012); this can also affect vaccine responses (Malhotra et al., 2015). Similarly, children born to HIV-infected mothers, but not themselves infected, suffer higher morbidity and mortality (Slogrove et al., 2012, 2017; Moraleda et al., 2014; Rupérez et al., 2017; Goetghebuer et al., 2018). This is a high concern for public health since the successful implementation of measures to prevent mother-to-child transmission of HIV has resulted in a reduced incidence of pediatric HIV, but an increase in the number of HIV-exposed uninfected (HEU) infants, particularly in sub-Saharan Africa. In some of the sub-Saharan countries, up to 30% of pregnant women are HIV-infected (González et al., 2012). Biological causes behind the increased morbidity and mortality in HEU include, lower transfer of maternal antibodies to newborns, alterations in the immune system of infants due to exposure to antiretroviral drugs, the immune activation in the mother driven by the infection and also HIV exposure *in utero* (Ruck et al., 2016). Adverse birth outcomes in HEU such as prematurity (Et et al., 2004; Chen et al., 2012) and fetal anemia (González et al., 2017) may also negatively affect the newborn’s health outcomes.

The immunology behind the reduced HEU health outcomes is not clear, but studies comparing HEU with unexposed (HUU) children have shown an altered immune response (Abu-Raya et al., 2016; Evans et al., 2016). Innate immune responses are an essential defense against infectious agents in newborns and direct and shape the adaptive immune response. Importantly, innate responses can also contribute to immunopathology. The innate immune system senses microbial pathogens through pattern recognition receptors (PRR), such as toll-like receptors (TLR) and nucleotide-binding oligomerization domain (NOD)-like receptors, which recognize conserved pathogen-associated molecular patterns. Innate immunity can present immunological memory, i.e., initial stimulation can lead to enhanced (trained immunity) or diminished innate responses (tolerance) to the same or different stimulus (Netea et al., 2011). Previous studies have shown that PRR-mediated innate immune responses differ between neonates, infants and adults (Kollmann et al., 2012; Georgountzou and Papadopoulos, 2017). Most studies show that upon *in vitro* stimulation of cord blood with TLR ligands compared to adult peripheral blood, lower levels of pro-inflammatory cytokines such as TNF and IL-1β and higher levels of the anti-inflammatory cytokine IL-10 are induced (Kollmann et al., 2012; Georgountzou and Papadopoulos, 2017). Also, responses of IFN and T_*H*_1-supporting cytokines such as IL-12p70 are weaker in cord than adults, whereas responses of the T_*H*_17-promoting cytokines IL-6 and IL-23 are enhanced.

Differences in the innate system of HEU vs. HUU infants have been found. Overall, a pro-inflammatory and activated immune profile has been described in HEU infants (Lohman-Payne et al., 2018; Dirajlal-Fargo et al., 2019) with changes in the proportion and profile of innate cell subsets (European Collaborative Study, 2004; Bunders et al., 2005; Velilla et al., 2008). To our knowledge, very few studies have assessed innate responses in HEU infants. We previously found higher TNF, IL-6, and IL-12 concentrations in myeloid dendritic cells (DC) and monocytes in response to bacterial PRR ligands in South African children, particularly at 2 and 6 weeks of age, but no differences in plasmacytoid DC (Reikie et al., 2013). However, other studies reported a reduced IL-12 production by cord monocytes in HEU infants (Chougnet et al., 2000) and no IL-6 responses upon TLR4 stimulation in some HEU infants (Maloupazoa Siawaya et al., 2018). Another study showed no differences upon TLR9 stimulation on DC (Velilla et al., 2008).

Despite evidence of the impact of maternal condition on the infant’s health, there is limited data on the relationship between the innate responses in the pregnant woman and their infants. Specifically, maternal cytokines may represent a stronger determinant of child immune responses than genetic factors (Djuardi et al., 2016). The extent to which innate immune responses to PRR differ or correlate between mothers and their newborns and how innate responses are altered by HIV exposure and other maternal biological factors such as anemia has not been well characterized. Here we aimed to (i) compare TLR- and NOD-mediated innate cytokine responses in HIV infected and uninfected women (peripheral and placental blood) and their infants (cord blood), (ii) estimate the association of HIV infection, maternal factors (age, gravidity, and anemia), and fetal sex with the innate cytokine profile, and (iii) explore the association of innate responses with birth outcomes.

## Materials and Methods

### Study Design

This is an observational study that included 48 women-cord pairs at the time of delivery. Study women were randomly selected among those recruited at the antenatal care clinic (ANC) in the Manhiça District Hospital (Mozambique) for an immunology study ancillary to two clinical trials comparing: (a) two-dose Intermittent Preventive Treatment in pregnancy (IPTp) with mefloquine (MQ) vs. two-dose IPTp-sulphadoxine-pyrimethamine (SP) in HIV-uninfected women (González et al., 2014b) and (b) three-dose IPTp-MQ and daily cotrimoxazole (CTX) prophylaxis vs. three-dose ITPp-placebo and daily CTX in HIV-infected women (González et al., 2014a; Supplementary Figure S1). The intensity of malaria transmission at the time of the study (2011–2013) was low/moderate (Mayor et al., 2015). All study women received bed nets treated with long-lasting insecticide, folic acid and ferrous sulfate. At the time of the study, antiretroviral therapy (ART) was recommended when CD4 + T cell count was below <350 cells/μL and/or when the woman was in 3 or 4 HIV/AIDS WHO clinical stage (WHO, 2005). ART was delivered to pregnant women at the monthly ANC visits. Prevention of mother-to-child transmission of HIV was based on antenatal administration of daily monotherapy with zidovudine (AZT) to the mother from 14 weeks of gestation, and combined antiretrovirals (single-dose nevirapine [NVP] and daily AZT plus lamivudine [3TC]) during labor and up to 1 week postpartum.

Participant characteristics and maternal and birth outcomes were recorded during the two clinical trials as described previously (González et al., 2014a, b). At delivery, blood samples (peripheral, placental, and cord blood) were collected into 10 mL Sodium Heparin tubes (BD Vacutainer, Cat No. 368480). Cord blood was collected from the umbilical arteries and placental blood was obtained from small incisions (1–1.5 cm-deep and long incisions) on the maternal-facing side of the placenta. Thick and thin blood films, as well as placental-biopsy samples (stored in 10% buffered formalin) were assessed for detection of *Plasmodium* species according to standard procedures (González et al., 2014a, b). Tissue samples from the maternal side of the placenta, as well as 50 μl of maternal peripheral, placental, and cord blood samples on filter papers, were collected for detection of *Plasmodium falciparum* in duplicate by means of a real-time quantitative polymerase-chain-reaction (qPCR) assay targeting 18S ribosomal RNA (Mayor et al., 2009). A capillary blood sample was collected from the infant at 6 weeks of age onto filter paper for HIV PCR analysis, following national guidelines for prevention of mother-to-child transmission of HIV. All newborns from whose blood we analyzed in the study were HIV-uninfected.

### Pattern Recognition Receptor Stimulations

Blood samples were processed in less than 4 h from collection. Whole blood was mixed 1:1 with sterile pre-warmed (37°C) RPMI 1640 medium. Two hundred microliters were added to each well of pre-made 96-well round-bottom polystyrene plates containing 22 μl of specific TLR and NOD ligands: PAM3CSK4 (PAM, TLR2/1); polyinosinic-polycytidylic acid (poly I:C, TLR3); lipopolysaccharide (LPS, TLR4); resiquimod (R848, TLR7/8); peptidoglycan (PGN, NOD1/2) and muramyl dipeptide (MDP, NOD2); and media alone. All ligands were diluted in RPMI medium to obtain the desired concentration: PAM (InvivoGen, San Diego, CA, United States) at 1 μg/mL; Poly I:C (GE Healthcare, Fairfield, CT, United States) at 100 μg/mL; LPS (InvivoGen) at 10 ng/mL; R848 (InvivoGen) at 10 μM; PGN (InvivoGen) at 10 μg/mL; MDP (InvivoGen) at 0.1 μg/mL. To standardize the assays, the pre-made plates were prepared, sealed with aluminum plate sealer and stored at −80°C until use.

The diluted whole blood was incubated for 24 h at 37°C in 5% CO_2_. After 24 h in culture, plates were centrifuged, 100 μL of supernatant were taken and stored at −80°C. The samples were shipped on dry ice via World Courier to Vancouver (Canada) where they were stored at −80°C until Luminex and ELISA-based measurements.

### Cytokine Quantification

The Luminex assay was performed in 26 Luminex plates and in two different phases some weeks apart. Supernatants from the 24 h culture plates were diluted 1:2 and 1:150 with RPMI and assessed in single replicates. Cytokines were assessed using the 13-plex Millipore Milliplex Map Kit (MPXHCTYO-60K): IFN-α2, IFN-γ, IL-1β, IL-6, IL-8, IL-10, IL-12p40, IL-12p70, IP10, MDC, MIP-1α, MIP-1β, and TNF-α. Manufacturer’s instructions were followed and the controls included in the kit were used. Data were analyzed in MiraiBio Masterplex QT. A 5-parameter logistic plot was used to calculate the standard curve and the sample concentrations. The lower and upper limits of detection were set as the lowest and highest concentration of the standard curve, respectively. The sample dilution factors were accounted for in setting the upper limit of detection. Samples with values below the detection limit for a specific cytokine were assigned a value half of the detection limit. When pooling the data of different plates, the higher lower limit of detection was applied for all plates. For a given sample and analyte, if readings were less than 50 beads, values were discarded; also, samples that had all analytes for all stimulations below the lower limit of detection were excluded from the analysis as this indicated failure of the biological assay. Twenty-five microliters of supernatants were used to also measure IL-23 in single replicates using the eBioscience Human IL-23 ELISA Ready-SET-Go kit (88-7237-86). Limits of detection were set as above. We selected these 14 cytokines because they cover key functional categories: cytokines supporting T_*H*_1-responses (IFN-α, IFN-γ, IP10, IL-12p70), cytokines supporting T_*H*_17 cytokines (IL-12p40, IL-6, and IL-23), pro-inflammatory cytokines (TNF and IL-1β) and chemokines (IL8, MIP-1α, MIP-1β, MDC) and the anti-inflammatory cytokine IL-10.

### Statistical Analysis

Descriptive analysis comparing characteristics HIV-uninfected and HIV-infected groups was performed using the compareGroups R package (Subirana et al., 2014). The compareGroups function performs a Shapiro–Wilk test for normality to decide if the variables are normal or non-normal distributed performing subsequently parametric or non-parametric tests, respectively.

Unsupervised analysis to visualize factors affecting variability in the cytokine concentration was performed using principal component analysis (PCA) with the FactoMineR package (Lê et al., 2008). Crude cytokine concentration values were used for the PCA and the three first components that explained most of the data variability were selected. A technical batch effect (effect of the phase in which Luminex plates were assayed) was detected and all analyses were performed by multivariable linear and logistic regressions adjusting by assay phase (Supplementary Figure S2C). Age was detected as one of the main factors influencing cytokine responses by univariable analyses and therefore was considered a confounding factor and was also used to adjust all analyses in multivariable models. Multivariable models were not adjusted for additional variables due to the limited sample size.

The analysis of cytokine responses to the PRR (agonist-specific cytokine responses) was performed with ratios of the concentrations of the stimulations divided by the unstimulated control (background). Concentration values from the unstimulated controls, considered spontaneous cytokine expression, were also analyzed. Ratios and concentration values were log_10_-transformed. Differences of crude cytokine concentrations between agonists and the unstimulated control were assessed by one-sided Wilcoxon signed-rank tests and differences between the three compartments (placental, cord, and peripheral) were assessed by Friedman tests.

Fold-change differences in cytokine responses in pairwise comparisons between compartments were assessed by multivariable linear regression analysis with cytokine ratios or concentrations as the dependent variable. Correlations of cytokine concentrations and ratios between different compartments were performed by Spearman. The association of gravidity (primigravidae [first pregnancy] vs. multigravidae [≥2 pregnancies], sex, maternal anemia at delivery [hemoglobin < 11 g/dL], HIV infection and being on ART at study baseline with cytokine responses was assessed separately by different multivariable linear regression models, with each factor as independent variable, age as a co-variable and cytokine ratio or concentration as the dependent variable. The relationship of cytokine responses with pregnancy outcomes (birth weight, low birth weight [<2500 gr], fetal hemoglobin, fetal anemia [<12.5 g/dL in cord blood], gestational age measured by Ballard score (Ballard et al., 1991) and prematurity [<37 weeks of gestational age] was assessed through multivariable logistic regressions (one for each factor separately), with cytokine response as independent variable. These multivariable models were also adjusted by age and batch effect.

*P*-values were adjusted for multiple testing by Benjamini–Hochberg (False Discovery Rate, FDR); the number of tests performed for each outcome was stated in the respective tables’ footnote. Adjustments of *p*-values were performed separately for each maternal variable and birth outcome to allow the assessment of each variable independently. Due to the exploratory nature of this study we chose to consider adjusted *p*-values ≤ 0.3 (FDR 30%) as significant. All analyses were performed in R software version 3.5 and 3.6.0 (R Core Team, 2019). Additional packages used for data management were reshape2, tidyverse and dplyr (Wickham, 2007, 2016b; Wickham et al., 2015). The package ggplot2 (Wickham,2016a) was used for all boxplots, heatmaps, and forestplots.

## Results

### Baseline Characteristics of Study Participants

A total of 48 women (35 HIV-uninfected and 13 HIV-infected) with peripheral, placental and corresponding cord blood samples available were included in the study. All HIV-uninfected women participated in a clinical trial comparing MQ to SP as IPTp (González et al., 2014b). Most of them (*N* = 33) received MQ whereas only 3 received SP (Table 1 and Supplementary Figure S1). All HIV-infected women participated in the trial comparing MQ plus daily CTX vs. only CTX (González et al., 2014a) and only 3 received MQ (Table 1 and Supplementary Figure S1). Therefore, the associations with the different IPTp treatments were not assessed. The median age of the women was 22 years, 33.3% of them were primigravidae, 33.3% had anemia and 43.8% gave birth to a female baby (Table 1). Based on the middle-upper arm circumference (MUAC), only one HIV-uninfected and one infected woman had malnutrition (MUAC < 22 cm). There were five cesarean deliveries. Regarding birth outcomes, 6.3% of the newborns had fetal anemia, 8.3% had low birth weight, and 16.7% were premature. There were no significant differences between HIV-uninfected and HIV-infected women with the exception of fetal hemoglobin levels in the cord, which were significantly lower in those born to HIV-infected women. HIV PCR of all study infants was negative at 6 weeks of age.

**TABLE 1:** Summary descriptive of study population by groups of HIV.

	[All] *N* = 48	HIV-uninfected *N* = 35	HIV-infected *N* = 13	*P*-value
**Age,** median[Q1;Q3]	22.0 [18.8;27.0]	21.0 [17.5;26.5]	25.0 [21.0;27.0]	0.153^a^
**Gravidity,** N(%)**:**				0.170^b^
**MG**	32 (66.7%)	21 (60.0%)	11 (84.6%)	
**PG**	16 (33.3%)	14 (40.0%)	2 (15.4%)	
**Fetal sex,** N(%)**:**				0.437^b^
**female**	21 (43.8%)	17 (48.6%)	4 (30.8%)	
**male**	27 (56.2%)	18 (51.4%)	9 (69.2%)	
**Maternal hemoglobin at delivery,** mean (SD)	11.2 (1.52)	11.3 (1.69)	11.0 (0.93)	0.436^c^
**Maternal anemia at delivery (hemoglobin < 11 g/dL),** N(%)**:**				0.735^b^
**no**	32 (66.7%)	24 (68.6%)	8 (61.5%)	
**yes**	16 (33.3%)	11 (31.4%)	5 (38.5%)	
**Fetal hemoglobin,** mean (SD)	14.2 (1.24)	14.4 (1.23)	13.5 (1.00)	0.011^c^
**Fetal anemia (<12 g/dL in cord blood),** N(%)**:**				0.174^b^
**no**	45 (93.8%)	34 (97.1%)	11 (84.6%)	
**yes**	3 (6.3%)	1 (2.7%)	2 (15.4%)	
**Birth weight,** median[Q1;Q3]	3050 [2900;3300]	3100 [2900;3250]	3000 [2900;3300]	0.692^a^
**Low birth weight (<2500 gr),** N(%)**:**				0.294^a^
**no**	44 (91.7%)	33 (94.3%)	11 (84.6%)	
**yes**	4 (8.3%)	2 (5.7%)	2 (15.4%)	
**Gestational age,** median[Q1;Q3]	38.0 [37.0;39.0]	38.0 [37.0;39.0]	38.0 [37.0;39.2]	0.601^a^
**N missings**	4	3	1	
**Prematurity (<37 weeks),** N(%)**:**				1.000^b^
**no**	40 (83.3%)	29 (82.9%)	11 (84.6%)	
**yes**	8 (16.7%)	6 (17.1%)	2 (15.4%)	
**MUAC (cm) at recruitment,** mean (SD)	25.6 (2.16)	25.7 (2.16)	25.3 (2.21)	0.531^c^
**N missings**	1	–	1	
**Mode of delivery,** N(%)**:**				1.000^b^
**Normal vaginal**	42 (89.4%)	31 (88.6%)	11 (91.7%)	
**Cesarean section**	5 (10.6%)	4 (11.4%)	1 (8.3%)	
**N missings**	1	–	1	
**CD4+ T cell counts at delivery,** mean (SD)	–	–	837 (580)	na
**HIV viral load at delivery,** median[Q1;Q3]	–	–	1116 [1.00;20457]	na
**IPTp,** N(%):				na
**2-dose MQ**	–	32 (91.4%)	–	
**2-dose SP**	–	3 (8.6%)	–	
**3-dose MQ + daily CTX**	–	–	3 (23.1%)	
**Placebo + daily CTX**		–	10 (76.9%)	

*^*a*^Non-parametric test. ^*b*^Chi-square. ^*c*^Parametric test. CTX, cotrimoxazole; IPTp, Intermittent Preventive Treatment in pregnancy; MG, multigravidae; MQ, mefloquine; MUAC, middle upper arm circumference; na, not applicable; PG, primigravidae; Q, quartile; SD, standard deviation; SP, sulphadoxine-pyrimethamine.*

Six out of 13 HIV-infected women received ART before pregnancy. None of the study women had placental malaria and all had negative *P. falciparum* blood smears during ANC visits; parasites were detected by qPCR in 5 out of 43 study women at recruitment. No submicroscopic data was available for the other two visits and therefore associations with malaria were not assessed in this study.

### Innate Cytokine Profile

We initially explored all cytokine data in an unsupervised approach using PCA that allows the reduction of the dimensionality of data into a new set of uncorrelated variables (principal components). Supplementary Figure S2A shows the PCA scores of all maternal peripheral, placenta and cord blood samples by the 6 PRR agonists using the first three dimensions. Only pI:C and R848 had a clear differential profile from the unstimulated control. Despite no clear clustering in the PCA, cytokine levels still differed significantly from the unstimulated control for all PRR agonists for most cytokines (Supplementary Figure S3). R848 showed the highest responses while MDP was the weakest stimulant with no statistically significant differences in IL-23, IL-12p70, IP10, and MDC concentrations compared to the unstimulated control. Therefore, we used the cytokine concentrations in the unstimulated control sample and all the agonists-specific cytokine responses for the analysis. The agonist-specific responses were analyzed for each cytokine using ratios between the cytokine concentration in the agonist stimulation and the cytokine concentration in the unstimulated control (Supplementary Figure S4).

No clustering of data was observed for blood compartment (periphery, cord, placenta, Supplementary Figure S2B) or any other factor in the PCA analysis, reflecting that samples did not differ substantially in their cytokine profile despite being from different compartments and coming from mothers with diverse characteristics. The Luminex assays were performed in two phases which we detected to affect the cytokine data (Supplementary Figure S2C), therefore the phase in which the assays were performed was taken into account in the subsequent analyses adjusting the models by phase.

### Comparison of Cytokine Levels Between Compartments

**FIGURE 1:**
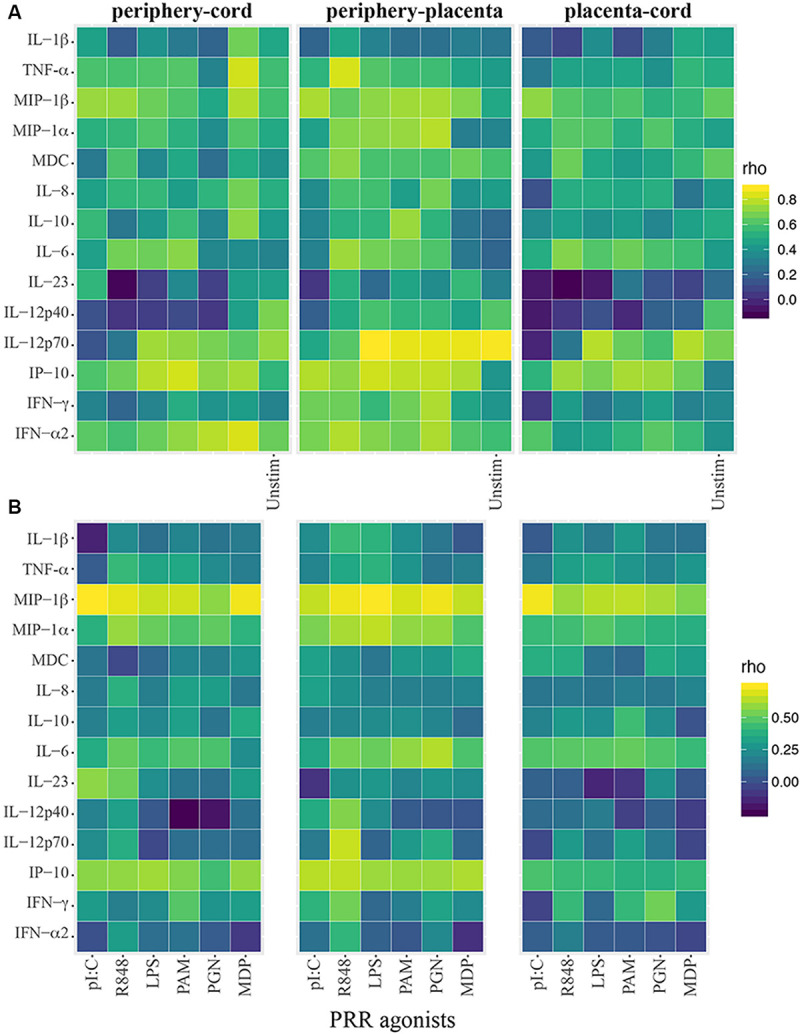
Heatmaps showing innate cytokine correlations between cord and periphery and placenta. **(A)** Spearman correlation coefficients (rho) using crude cytokine concentrations upon innate stimulations or spontaneous production of cytokines (background control = Unstim). **(B)** Spearman correlations using agonist-specific cytokine responses expressed as ratios of cytokine production of innate stimulations over background control.

Overall cytokine production was positively correlated between the different compartments, especially between maternal peripheral blood and cord blood (Figure 1A). In particular, spontaneous production of TNF and MIP-1β, IL-12p40, IL-12p70, and IFN-α was highly correlated between those two compartments (rho = 0.58–0.73). Regarding the cytokine responses to PRR agonists (Figure 1B), MIP1-α, MIP-1β, and IP10 were strongly correlated in peripheral and cord blood for all agonists. Some other high positive cytokine correlations were found but varied between agonists. In general, spontaneous production of cytokines and agonist-specific responses in cord blood were also positively correlated with the ones in the placental blood (Figure 1A), similarly to correlations between cord and periphery, but weaker.

**FIGURE 2:**
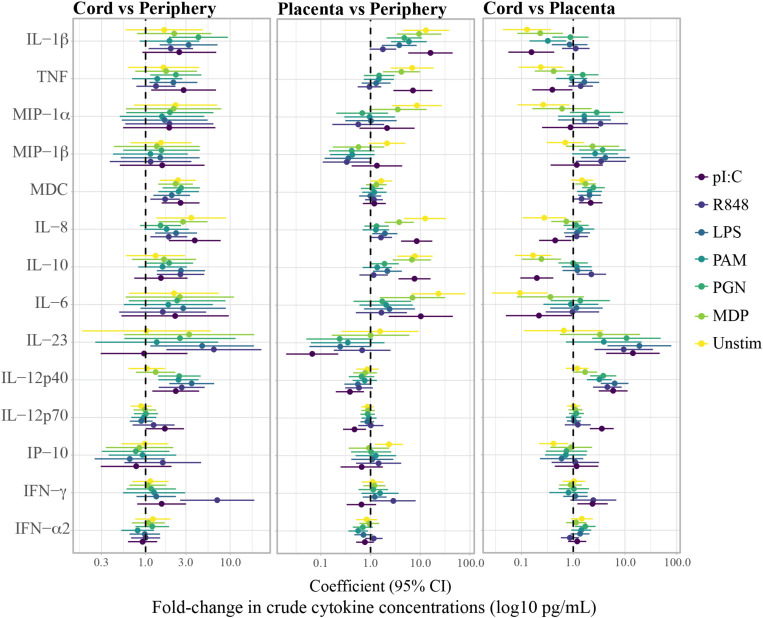
Fold-change differences between compartments in crude cytokine concentrations upon whole blood stimulations with agonists of pattern recognition receptors. Forest plots showing the effect (coefficients and 95% confidence intervals) of cord vs. periphery, cord vs. placenta and placenta vs. periphery, on cytokine concentrations. Coefficients were calculated as 10^beta and beta was obtained by multivariable regressions adjusted by age (log_10_-transformed) and technical batch, with cytokine concentrations (log10-transformed) as outcome.

**FIGURE 3:**
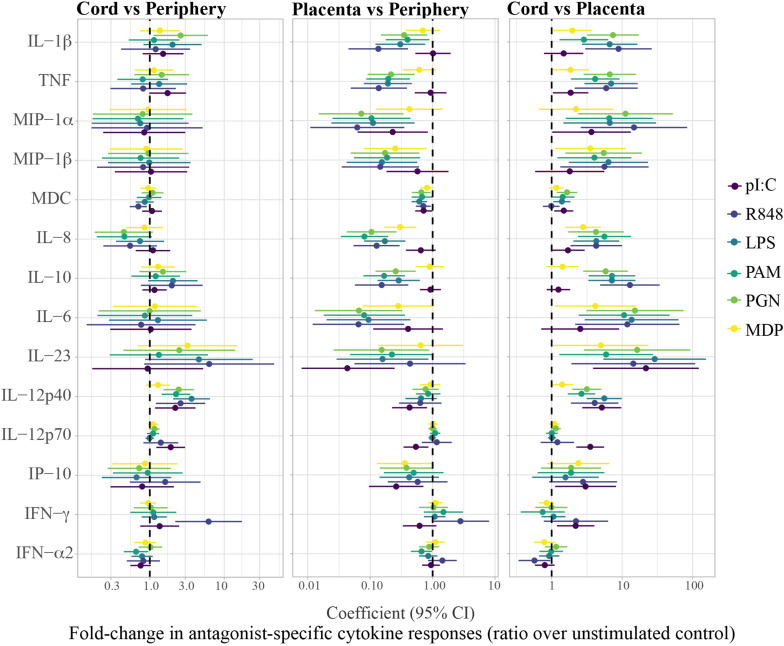
Fold-change differences between compartments in agonist-specific cytokine responses (ratios). Agonist-specific cytokine responses are expressed as ratios of cytokine concentrations upon stimulation over background control (unstim). Forest plots showing the effect (coefficients and 95% confidence intervals) of cord vs. periphery, cord vs. placenta and placenta vs. periphery, on cytokine responses. Coefficients were calculated as 10^beta and beta was obtained by multivariable regressions adjusted by age (log_10_-transformed) and technical batch, with cytokine ratios (log10-transformed) as outcome.

Cytokine concentrations in the unstimulated control and in the PRR stimulations differed among periphery, placenta and cord, particularly for the pro-inflammatory cytokines and chemokines, the anti-inflammatory cytokine IL-10 and the T_*H*_17 cytokines IL-6 and IL-12p40 (Supplementary Figure S5). Figures 2, 3 show the fold-change difference between compartments in the crude cytokine concentrations (Figure 2) and the agonist-specific cytokine responses (Figure 3), calculated using adjusted models. Overall, cytokine concentrations were higher in cord than in maternal peripheral blood. Specifically, spontaneous production of the pro-inflammatory MDC and IL-8 chemokines was 2.4 and 3.5 times higher, respectively, in cord than in peripheral maternal blood (Figure 2). Also, IL-12p40 production for all PRR with the exception of MDP, IL-1β for NOD1/2, IL-12p70 for TLR3 and IFN-γ for TLR7/8 were higher in cord (Figure 3), but lower for MDC in response to TLR7/8 and for IFN-α in response to TLR1/2 stimulation (Figure 3). Spontaneous production of cytokines in placenta was higher than in periphery and cord for the pro-inflammatory cytokines IL-1β, TNF, MIP-1α, and IL-8, the anti-inflammatory IL-10, the T_*H*_17 cytokine IL-6 and the T_*H*_1 cytokine IP10 (Figure 2). However, there were diminished PRR agonist-specific responses in placental blood compared to the other two compartments (Figure 3). Therefore, despite a general positive correlation of innate cytokine responses between compartments, some differences were found in cord compared to peripheral maternal blood.

### Association of HIV-Infection and Other Maternal Factors With Cytokine Responses

**FIGURE 4:**
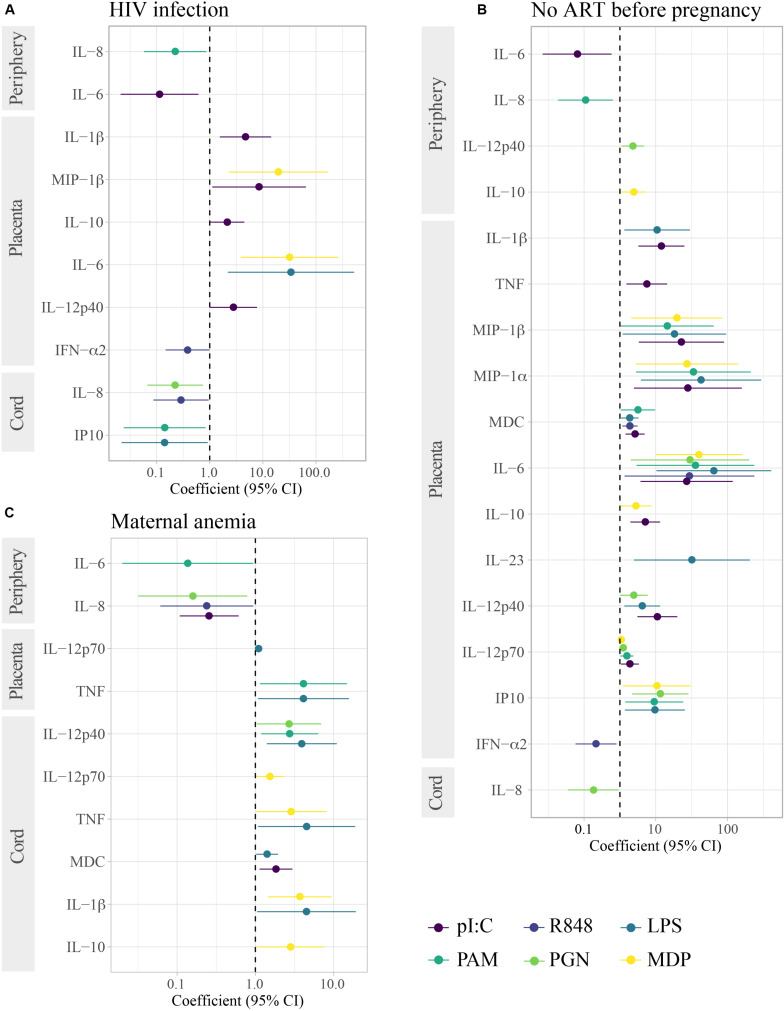
Association of HIV infection, lack of ART before pregnancy and maternal anemia with cytokine responses induced by innate stimulations. Forest plots show the effect of **(A)** HIV-infected in reference to HIV-uninfected women, **(B)** lack of ART before pregnancy in HIV-infected women in reference to HIV-uninfected women, and **(C)** maternal anemia in reference to no anemia in HIV-infected and uninfected women. Only results with raw *p*-values < 0.05 are shown. All results in **(C)** were statistically significant after adjusting for multiple testing by Benjamini–Hochberg. The coefficient was calculated as 10^beta and beta was obtained in multivariable regression models adjusted by age (log_10_-transformed) and technical batch with cytokine ratios (log_10_-transformed) as outcome.

HIV-exposure was not associated with spontaneous production of cytokines (Table 2). For some PRR stimulations, particularly TLR3, HIV-infected compared to HIV-uninfected mothers, produced less IL-6 and IL-8 in peripheral blood, yet much higher IL-1β, MIP-1β, IL-10, IL-12p40, and IL-6, but less IFN-α (for TLR7/8) in placental and lower IL-8 and IP10 in cord blood (Figure 4). However, these associations were only statistically significant after adjusting for multiple testing when analyzing HIV-infected women who did not receive ART prior to pregnancy (Figure 4).

**TABLE 2:** Association of HIV infection, gravidity, infant’s sex and maternal anemia with spontaneous cytokine production.

	Compartment	Cytokine	Cytokine group	Coefficient^a^	95% CI	*P*-value^b^	BH *P*-value^c^
**HIV infection (ref: HIV-uninfected)**							
	Cord	IL-12p70	TH1	0.648	0.429; 0.979	0.04	0.993
**Gravidity (ref: multigravidae)**							
	Periphery	MIP-1α	Pro-inflammatory	0.068	0.01; 0.471	0.008	0.28
	Periphery	MIP-1β	Pro-inflammatory	0.158	0.034; 0.729	0.019	0.28
	Periphery	IL-23	TH17	0.018	0.001; 0.509	0.02	0.28
	Cord	IP10	TH1	0.312	0.103; 0.947	0.04	0.993
**Sex (ref: females)**							
	Periphery	IL-1β	Pro-inflammatory	0.243	0.065; 0.914	0.037	0.389
	Periphery	IL-8	Pro-inflammatory	0.211	0.05; 0.895	0.035	0.389
	Periphery	IFN-α2	TH1	0.35	0.163; 0.752	0.008	0.21
	Periphery	IL-6	TH17	0.127	0.027; 0.601	0.01	0.21
**Maternal anemia (ref: no anemia)**							
	Periphery	IL-8	Pro-inflammatory	5.984	1.315; 27.234	0.022	0.651
	Periphery	IL-6	TH17	6.433	1.193; 34.679	0.031	0.651
	Cord	IFN-γ	TH1	0.432	0.188; 0.993	0.048	0.672

*Only results with raw *p*-values ≤ 0.05 are shown. ^*a*^Coefficients are 10^∧^beta and beta was obtained in multivariable regression models adjusted by age (log_10_-transformed) and technical batch with cytokine concentrations (log_10_-transformed) as outcome. ^*b*^Raw *p*-value. ^*c*^Significance was established at a Benjamini–Hochberg (BH) adjusted *p*-value ≤ 0.3. *P*-values were adjusted by a total of 42 tests performed for each independent variable.*

In analysis including HIV-uninfected and -infected women, higher maternal age was associated with lower spontaneous production of pro-inflammatory cytokines as well as the IL-10 anti-inflammatory cytokine in women’s peripheral blood (Supplementary Table S1). Age also was associated with higher cytokine production following PRR stimulation in peripheral blood when not adjusting for multiple comparisons (Supplementary Table S2). As age was the non-clinical variable with the largest association with cytokine responses, we adjusted all analyses, including the above, by age.

Gravidity was also associated with spontaneous production of cytokines in maternal peripheral blood: Primigravidae had lower spontaneous production of MIP-1α, MIP-1β, and IL-23 (Table 2). Upon stimulation though, primigravidae produced more IL-10, IL-8, TNF, and IL-23 in maternal peripheral blood, but less IL-1β in placental and less IFN-γ in cord blood (Supplementary Figure S6). However, these later results were not statistically significant after adjusting for multiple testing.

Fetal sex had a remarkable impact on cytokine responses. Mothers who delivered a male vs. female newborn exhibited lower spontaneous production of IL-1β, IL-8, IL-6, and IFN-α in their peripheral blood (Table 2), although associations of the first two were not significant after adjusting for multiple testing. It is worth mentioning that male vs. female newborns had lower IP10 responses for all PRR stimulations despite lack of statistical significance after adjusting for multiple testing (Supplementary Figure S6).

When considering raw *p*-values < 0.05, maternal anemia was associated with higher spontaneous production of IL-8 and IL-6 in peripheral blood and lower production in cord blood of IFN-γ (Table 2). In contrast, upon PRR stimulation (Figure 4), anemic women had lower IL-8 and IL-6 responses. Maternal anemia was also associated with cytokine responses in cord (particularly for MDP and LPS), with higher IL-10, IL-1β, MDC, TNF, IL-12p70, IL-12p40 responses following PRR stimulation.

In summary, HIV infection when ART was not administered before pregnancy, affected mainly placental blood responses, with little to no impact on maternal peripheral or cord blood responses. On the other hand, age and gravidity (independently from age) affected some spontaneous maternal peripheral responses, while maternal anemia associations did not reach statistical significance after adjusting for multiple testing. Curiously, fetal sex was associated with particular spontaneous peripheral maternal responses.

### Association of Innate Cytokines With Birth Outcomes

We did not find any statistically significant association of spontaneous production of cytokines with birth outcomes when adjusting for multiple testing (Supplementary Table S3). However, higher spontaneous production of IL-8 and IL-6 in maternal peripheral blood could be associated with lower fetal hemoglobin (raw *p*-value < 0.05). This is consistent with the trends found for maternal anemia and higher IL-8 and IL-6, considering that maternal anemia can be associated with fetal anemia. On the other hand, maternal peripheral blood PRR responses of IFN-γ were significantly associated with lower fetal hemoglobin, whereas IL-6 and IL-8 were associated with considerable increases (Table 3).

**TABLE 3:** Association of cytokines induced by innate stimulations with birth outcomes.

Compartment	Agonist	Receptor	Receptor localization	Cytokine ratio	Cytokine group	Coefficient (%)^a^	95% CI	*P*-value^b^	BH *P*-value^c^
**Birth weight**									
Periphery	R848	TLR7/8	Endosomal	IL-12p70	TH1	0.599	0.019; 1.181	0.043	0.983
Placenta	R848	TLR7/8	Endosomal	IL-12p40	TH17	0.366	0.036; 0.697	0.031	0.983
**Fetal hemoglobin**									
Periphery	MDP	NOD2	Cytosolic	IFN-γ	TH1	–32.054	−47.201; −12.561	0.004	0.294
Periphery	PAM	TLR1/2	Surface	IL-6	TH17	3.625	1.049; 6.267	0.007	0.294
Periphery	LPS	TLR4	Surface	IL-6	TH17	3.499	0.975; 6.086	0.007	0.294
Periphery	R848	TLR7/8	Endosomal	IL-6	TH17	2.594	0.307; 4.932	0.027	0.601
Periphery	R848	TLR7/8	Endosomal	IL-8	Pro-inflammatory	5.578	1.963; 9.321	0.003	0.294
Periphery	pI:C	TLR3	Endosomal	IL-8	Pro-inflammatory	8.297	2.803; 14.084	0.004	0.294
Periphery	PAM	TLR1/2	Surface	IL-8	Pro-inflammatory	5.675	1.731; 9.773	0.006	0.294
Periphery	PGN	NOD1/2	Cytosolic	IL-8	Pro-inflammatory	4.243	1.068; 7.517	0.01	0.360
Periphery	LPS	TLR4	Surface	IL-8	Pro-inflammatory	5.271	1.141; 9.569	0.013	0.410
Periphery	MDP	NOD2	Cytosolic	IL-8	Pro-inflammatory	6.625	1.011; 12.551	0.021	0.588
Periphery	PAM	TLR1/2	Surface	IP10	TH1	2.85	0.031; 5.749	0.048	0.657
Periphery	LPS	TLR4	Surface	MIP-1β	Pro-inflammatory	2.891	0.109; 5.75	0.042	0.657
Periphery	PAM	TLR1/2	Surface	MIP-1β	Pro-inflammatory	2.904	0.094; 5.794	0.043	0.657
Cord	PGN	NOD1/2	Cytosolic	IL-12p40	TH17	–5.078	−9.712; −0.207	0.042	0.657
Cord	PGN	NOD1/2	Cytosolic	IL-12p70	TH1	–13.054	−23.336; −1.393	0.03	0.601
Cord	pI:C	TLR3	Endosomal	IL-1β	Pro-inflammatory	–4.931	−9.182; −0.48	0.031	0.601
Cord	pI:C	TLR3	Endosomal	MDC	Pro-inflammatory	–10.339	−18.504; −1.356	0.026	0.601
**Gestational age**									
Placenta	R848	TLR7/8	Endosomal	IL-12p40	TH17	0.092	0.003; 0.182	0.044	0.837
Placenta	LPS	TLR4	Surface	IP10	TH1	–0.104	−0.179; −0.028	0.008	0.837
Placenta	MDP	NOD2	Cytosolic	IP10	TH1	–0.089	−0.159; −0.02	0.013	0.837
Placenta	PAM	TLR1/2	Surface	IP10	TH1	–0.094	−0.174; −0.014	0.023	0.837
Placenta	MDP	NOD2	Cytosolic	TNF	Pro-inflammatory	0.13	0.024; 0.236	0.018	0.837
Placenta	PAM	TLR1/2	Surface	TNF	Pro-inflammatory	0.094	0.006; 0.182	0.038	0.837
Cord	MDP	NOD2	Cytosolic	IL-8	Pro-inflammatory	–0.123	−0.225; −0.02	0.021	0.837

*Only results with raw *p*-values ≤ 0.05 are shown. ^*a*^Coefficients show the difference in percentage in the outcome with 10% increases in cytokine ratios. It was calculated as (1.10^beta-1) × 100 and beta was obtained in multivariable regression models adjusted by age (log_10_-transformed) and technical batch with cytokine ratios (log_10_-transformed) as predictor variable and birth outcomes as dependent variable. Birth weight and gestational age were log_10_-transformed. ^*b*^Raw *p*-value. ^*c*^Significance was established at a Benjamini–Hochberg (BH) adjusted *p*-value ≤ 0.3. *P*-values were adjusted by a total of 252 tests performed for each independent variable.*

Regarding newborn’s gestational age and prematurity, there were no statistically significant associations with cytokine responses (Table 3 and Supplementary Tables S3–S5). Nevertheless, consistent associations when considering raw *p*-values were detected: Ten-fold changes in spontaneous production of IL-8 in cord blood were associated with 0.337 odds of being premature (Supplementary Table S4), whereas IL-8 responses in cord blood were associated with lower gestational age (Table 3); and 10-fold increases in IL-8 responses to all PRR stimulations, with the exception of TLR1/2, were associated with 3.5–5 increased odds of prematurity (Supplementary Table S5).

## Discussion

Our study contrasting innate immunity across the three compartments of maternal peripheral blood, placental blood and newborn cord blood provides evidence in support of the notion that maternal HIV infection impacts innate responses in both the woman and the HIV-exposed but uninfected child. Given the unique access to the triad of maternal peripheral and placental blood as well newborn cord blood, we were able to identify that the relationship of maternal HIV-infection with cytokine responses was predominantly evident in the placental blood compartment. Furthermore, our study design also allowed us to identify that this impact of HIV on maternal and newborn innate immunity was restricted to women who had not received ART before pregnancy. This has substantial public health implications, as this change in immune status is presumed to be related to adverse birth outcomes such as low birth weight and prematurity, and thus all HIV-infected women should be on ART. Our results further reveal that other maternal factors could further influence innate responses, such as maternal age, gravidity and infant’s sex.

HIV-infected women who did not receive ART at baseline had decreased IL-6 and IL-8 responses to PRR stimulations in peripheral blood. HIV itself may activate TLR7/8 (Meier et al., 2007) and other PRR (Mogensen et al., 2010) contributing to the HIV chronic immune activation. In addition, systemic bacterial translocation caused by HIV infection (Brenchley et al., 2006) may also be activating TLR and promoting further immune activation. According to our results, higher baseline immune activation and TLR stimulation could be causing hyporesponsiveness or tolerance upon further stimulation, which we speculate could lead to increased susceptibility to infections. Our findings are consistent with previous studies showing a decreased response to TLR in HIV-infected individuals, which inversely correlated with viral load (Scully et al., 2016). However, other studies showed that responses depended on the cell subset and the HIV infection stage (Chang et al., 2012) and increased TLR expression and responsiveness with HIV infection have also been observed (Lester et al., 2008; Hernández et al., 2012). Of note, in these studies the response to TLR was measured in PBMC instead of whole blood and in different conditions than ours. In addition, all of the other studies were performed in non-pregnant adults, and sex (Meier et al., 2009) and pregnancy (Ziegler et al., 2018), which is characterized by immunoregulatory mechanisms to maintain the semi-allogeneic fetus, affect innate responses. To our knowledge, only one study assessed innate responses to TLR ligands in HIV-infected pregnant women and in their newborns (Cardoso et al., 2013). In that study and similarly to our results, a compromised cytokine (TNF, IFN-α, and IL-10) response to TLR was detected in both maternal and cord blood with the exception of TLR7/8 response in myeloid DC.

The associations of HIV infection when not receiving ART before pregnancy with placental PRR responses, suggest that HIV may be particularly affecting the placenta. We found that women who had not received ART before pregnancy had higher pro-inflammatory and anti-inflammatory IL-10 (which usually go hand in hand with pro-inflammatory cytokines), and T_*H*_1 and T_*H*_17 PRR responses in placental blood than HIV-uninfected women. The only exception worth noting was IFN-α in response to R848 stimulation, which was lower in HIV-infected than uninfected women. It would be of interest to further investigate how these responses may affect the fetus and if they could be associated with the adverse birth outcomes described in HIV-infected women (Chen et al., 2012). The increased impact of HIV-infection when not receiving ART before pregnancy is in line with recent results showing that ART initiation during pregnancy, instead of before, was associated with the activation of newborn monocytes, a reduced placental transfer of maternal antibodies and a higher risk of hospitalization of infants (Goetghebuer et al., 2018). Our findings support that initiation of ART before conception could benefit pregnancy and newborns, beyond prevention of perinatal transmission.

HIV exposed uninfected newborns had lower pro-inflammatory (IL-8) responses in cord blood compared to HUU newborns, although these differences were only statistically significant after adjusting for multiple testing when analyzing mothers not receiving ART before pregnancy. In our previous study, we had found higher IL-6, TNF, IL-12 responses in innate cells of HEU compared to HUU, but other cytokines besides IFN-α were not analyzed (Reikie et al., 2014). Moreover, in that study we did not assess concentrations in supernatants nor responses in cord. The diminished IL-8 PRR responses in cord in HEU could be of particular interest because of the consistent associations of IL-8 with gestational age and prematurity, although they were not statistically significant when adjusting for multiple testing.

Anemia during pregnancy is frequent, particularly in low-income countries, and HIV infection further increases the risk of anemia in pregnancy (González et al., 2017). There are multifactorial causes for the anemia, being malaria an important factor. However, none of the women of this study had microscopic or placental malaria. Our results were not statistically significant after adjusting for multiple testing, but were biologically plausible and consistent with the literature. Anemic mothers showed higher spontaneous production of pro-inflammatory IL-8 and the T_*H*_17 cytokine IL-6. Chronic inflammation is another of the factors causing anemia and IL-6 contributes to the inflammation and development of anemia through the iron regulatory hormone hepcidin, and the iron exporter ferroportin (Fraenkel, 2015). Furthermore, activation of TLR results in iron sequestration (Abreu et al., 2018). Although not significant after adjusting for multiple testing, we also found that anemia could be associated with lower responsiveness to PRR stimulations, similarly to what has been described in anemic children (Liao et al., 2018). While higher spontaneous production of IL-8 and IL-6 may reflect the pro-inflammatory status of the mothers, particularly the HIV-infected ones, and may be mediators of the development of anemia, the response to PRR suggest an impact of anemia on the immune response. Diminished IL-6 and IL-8 PRR responses observed in HIV-infected mothers who did not receive ART before pregnancy were associated with decreased fetal hemoglobin. Unfortunately, the interaction of HIV, maternal anemia and fetal anemia with the innate immune responses cannot be disentangled in our study and it is impossible to infer causality.

Other factors had some associations with the cytokine profile. Age is usually associated with cytokine responses and our study seems not to be an exception. Gravidity, which is related to age, was independently associated with lower spontaneous production of three cytokines on maternal peripheral blood. Curiously, sex of the newborn could be associated with the mother’s spontaneous cytokine production. Women carrying male fetuses had lower spontaneous production of T_*H*_1 and T_*H*_17 cytokines. Sexual dimorphism has been observed in maternal inflammation and cytokine profile in other studies (Stevenson et al., 2000; Enninga et al., 2015). Carrying a female fetus has been associated with more severe asthma in the mothers (Clifton, 2010) and fetal sex affects birth outcomes such as low fetal growth restriction, prematurity, and preeclampsia (Edwards et al., 2000). Sexual dimorphisms in the placenta have also been described (Clifton, 2010), but we did not find differences in placental blood in our study. Instead, in cord, IP10 responses in male infants for all PRR were lower than females, although these differences were not statistically significant after adjusting for multiple testing. Sex has a clear effect on immune response through life and particularly during infancy has been associated with different susceptibility to diseases and vaccine responses (Muenchhoff and Goulder, 2014; Fischinger et al., 2019). Future studies should assess if differences in IP10, a key chemokine in protective immunity and vaccine responses, are maintained through infancy and related to sex differences in clinical and vaccine outcomes.

It is not clear how maternal peripheral blood differs from placental or newborn cord blood, although cytokine responses in the mother seem to have a strong influence on cytokine responses in their infants (Djuardi et al., 2016). While overall cytokine concentrations and PRR responses were positively correlated between the mother peripheral and the placental blood and the cord blood, cytokine levels tended to be higher in cord blood stimulations than in peripheral blood, with some specific PRR responses higher in cord than in maternal blood. This is, apparently, in contradiction to previous reports showing a diminished T_*H*_1 and other responses in cord blood than adults (Kollmann et al., 2012; Georgountzou and Papadopoulos, 2017). We found only lower responses to IFN-α upon PAM in cord. However, here we compared cord blood responses to blood responses from their mothers at delivery instead of non-related non-pregnant adults. Of note, TLR7/8 responses were the most robust response as previously described (Kollmann et al., 2012; Georgountzou and Papadopoulos, 2017). Despite the coordinated innate response of the mothers with their children found here or in other studies (Djuardi et al., 2016), we did not find a clear association of peripheral blood cytokine responses with birth weight or gestational age and prematurity, only with fetal hemoglobin levels. Placental blood responses were less correlated with peripheral and cord responses and probably influenced by the delivery process which is known to be a pro-inflammatory placental process. In fact, we found higher spontaneous production of pro-inflammatory cytokines in the placenta. Due to the exposure of the placenta to the mothers’ microbiome during delivery and the procedure of sample collection, there is the potential risk of bacterial contamination in the placental blood. While that could be a problem because bacteria would stimulate PRR, we had unstimulated controls in the assay and our interpretation is in comparison of stimulated to those unstimulated samples. The finding that HIV may be associated specifically with placental responses and the association of some of the placental blood responses with gestational age and prematurity calls for further research on the immunopathology of this organ. The role of trophoblasts may be relevant, since these cells induce innate responses upon sensing of pathogens and orchestrate the recruitment and activation of innate cells at the maternal-fetal interface (Guleria and Pollard, 2000).

Despite some associations and other interesting findings of biological plausibility, the low sample size and the multiple tests performed demand interpreting the results with prudence, particularly because the potential for false positives is high. Findings should be confirmed with other techniques such ELISAs and additional cohorts. We measured secreted cytokines in whole blood, but particular cell subsets may be differently producing the measured cytokines upon PRR stimulation and therefore, further studies should include single-cell analyses. Also, it would be of interest to assess the expression of PRR in the cells of each compartment. Some findings may be specific from the study area and innate responses may vary in different geographic areas as has been shown in children from different continents (Smolen et al., 2014). Furthermore, inclusion of women participating in clinical trials may have posed some bias due to different treatments and additional medical attention that they may have received compared to other women in the area. We cannot discard either that the IPTp treatment received or the CTX administered to all HIV-infected women may have had an effect on the innate responses. Nevertheless, our data provide evidence of the association of HIV infection with innate responses to PRR in the mother, particularly obvious in the placenta. Results also suggest a complex relationship between HIV, maternal and fetal anemia and innate responses that may provide clues on anemia development. In addition, we report important data on specific innate responses correlating with birth outcomes that warrant follow up studies. Modulation of the innate response could be a powerful strategy to improve maternal and neonatal outcomes, but the causal association of innate responses with birth outcomes and infant’s health requires further detailed investigation.

## Data Availability Statement

All datasets generated for this study are included in the article/Supplementary Material.

## Ethics Statement

This study was carried out in accordance with ICH Good Clinical Practice guidelines and the Declaration of Helsinki. The study protocols and informed consent forms were reviewed and approved by the Comité Ètic d’Investigació Clínica (CEIC, Hospital Clínic, UB), Spain, and the Comité Nacional de Bioética (CNBS), Mozambique. Written informed consent was obtained from all participants.

## Author Contributions

GM, AM, CD, and TK wrote the first draft of the manuscript. TK, AM, CD, and GM conceived the study and the experimental design and interpreted the data. GM performed the statistical analysis. MM, RB, and CJ collected the samples and performed the stimulation experiments. KS performed the cytokine Luminex assay. RG, MR, JA, EM, and CM designed and enrolled participants in the clinical trials. JA was the clinical trial statistician. CD, RG, KS, CM, TK, and AM contributed to the write up of the manuscript. All authors reviewed and approved the manuscript.

## Conflict of Interest

The authors declare that the research was conducted in the absence of any commercial or financial relationships that could be construed as a potential conflict of interest.
