# Supplementary material for: Association of Maternal Factors and HIV Infection With Innate Cytokine Responses of Delivering Mothers and Newborns in Mozambique
Source: Front Microbiol. 2020 Jul 14;11:1452. doi: 10.3389/fmicb.2020.01452 (PMC7381182; doi:10.3389/fmicb.2020.01452)
Supplement: Supplementary file 3 [file Presentation_1.pdf]

Supplementary Material

1 Supplementary Data

Database of cytokine, demographic and clinical variables.

2 Supplementary Figures and Tables

2.1 Supplementary Figures

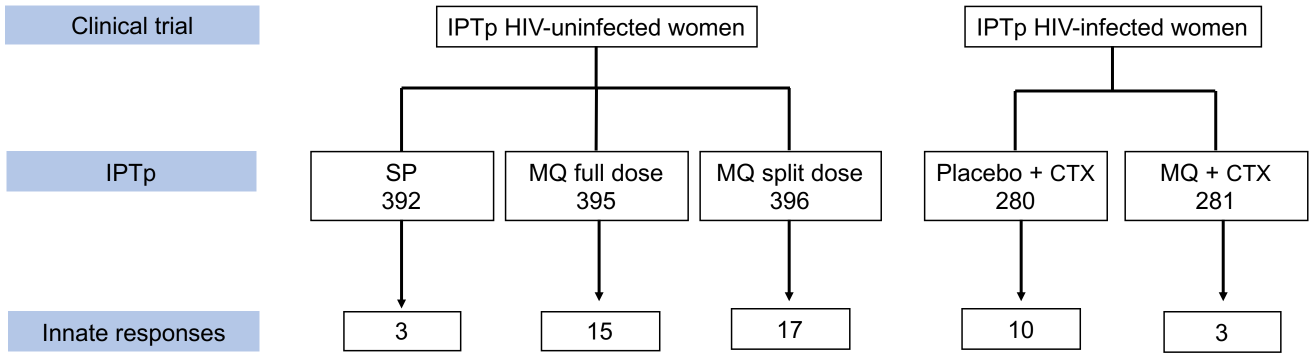

Supplementary Figure 1. Study flow chart. Study women (N=48) were randomly selected among those recruited at the antenatal care clinic in the Manhiça District Hospital (Mozambique) for two clinical trials testing IPTp drugs. The study performed in HIV-uninfected women had three arms: (1) SP, (2) single dose mefloquine (MQ,15 mg/kg), and (3) split-dose MQ. The study performed in HIV-infected women had two arms: (1) three-dose ITPp-placebo and daily CTX in HIV-infected women and (2) three-dose IPTp-MQ and daily cotrimoxazole (CTX) prophylaxis and.

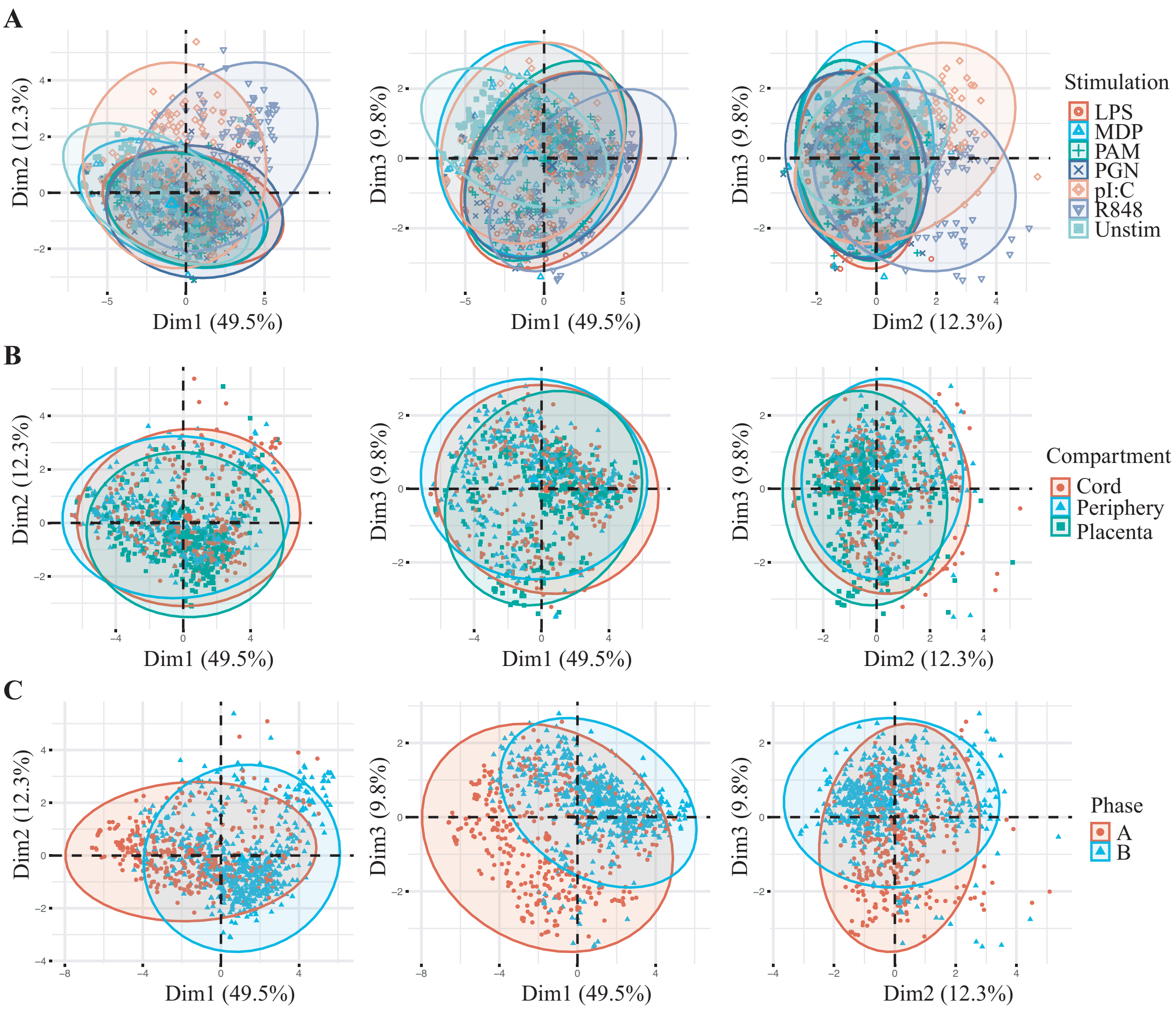

Supplementary Figure 2. Score plots of principal component analysis of innate cytokine profile. Plots of Dimensions (Dim) 1, 2 and 3 show the cytokine concentrations in all PRR agonist stimulations and the unstimulated control (A), in the 3 compartments (B) and by phase in which the assays were performed (technical batch) (C).

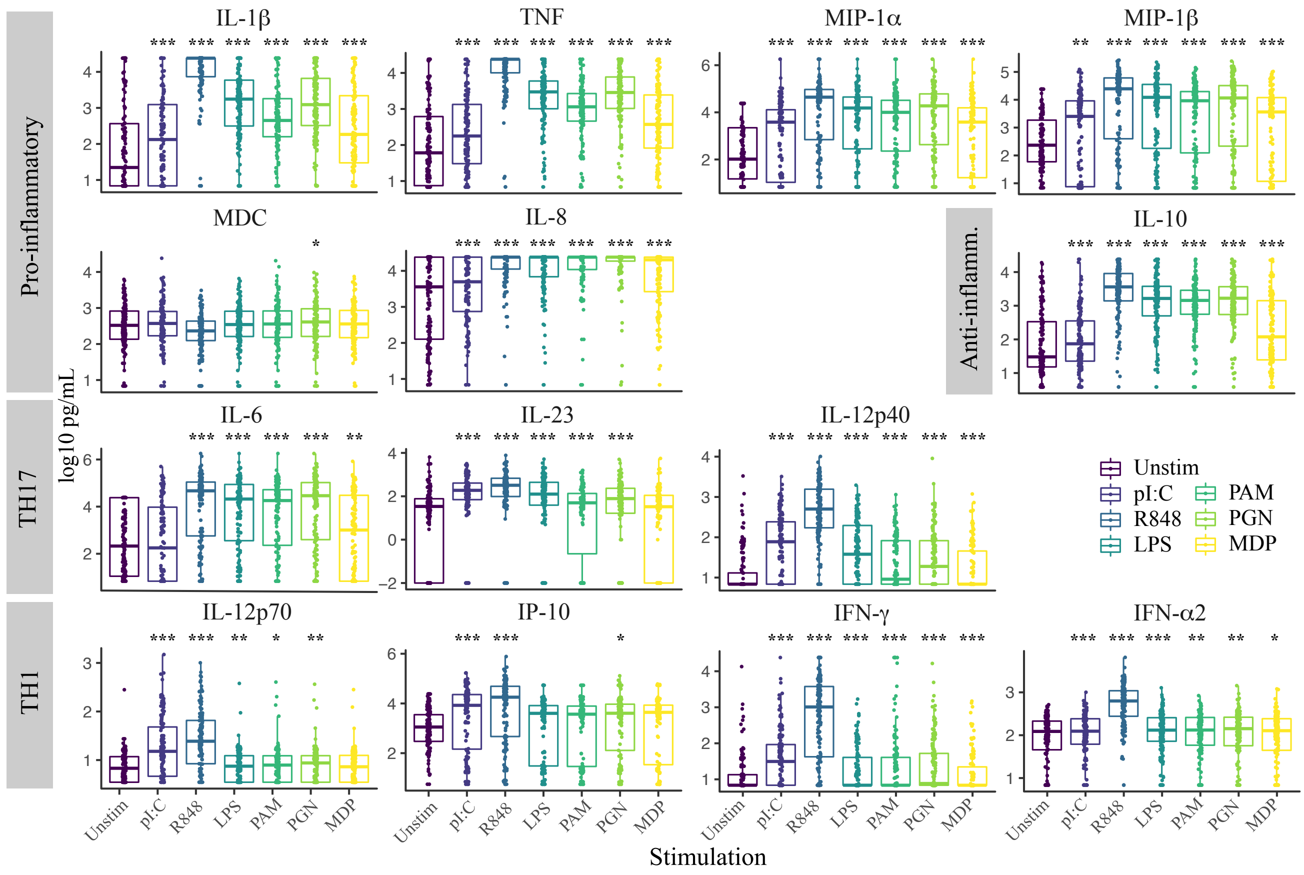

Supplementary Figure 3. Crude cytokine concentrations upon PRR agonist stimulations and the unstimulated control. Boxplots showing concentrations of different cytokines upon in vitro stimulations with different PRR agonists and the unstimulated control for all samples (peripheral, placental and cord blood). Differences between PRR agonists and the unstimulated control were assessed with one-sided Wilcoxon signed-rank tests (* p-value ≤ 0.05, ** p-value ≤ 0.01; *** p-value ≤ 0.001).

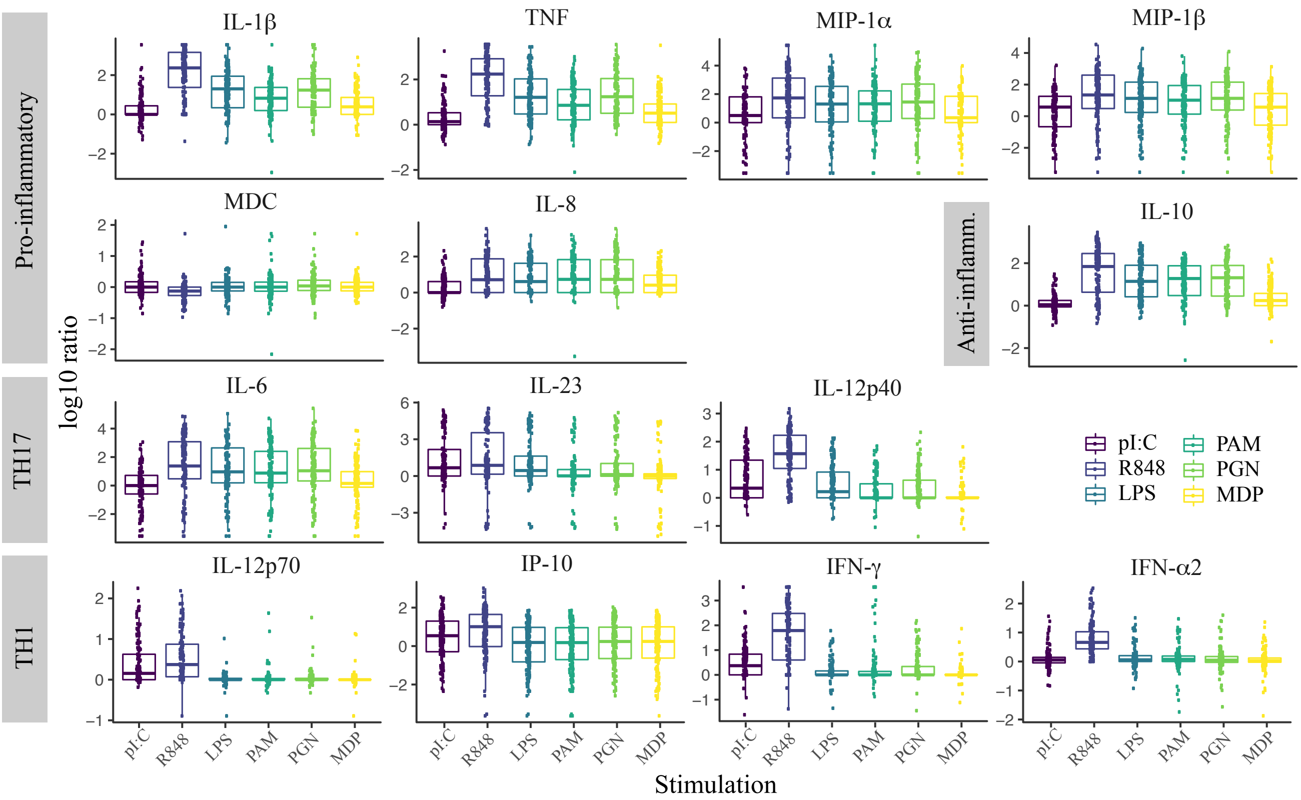

Supplementary Figure 4. Agonist-specific cytokine responses. Boxplots showing ratios of cytokines produced upon PRR agonists stimulations over background unstimulated control for all samples (peripheral, placental and cord blood).

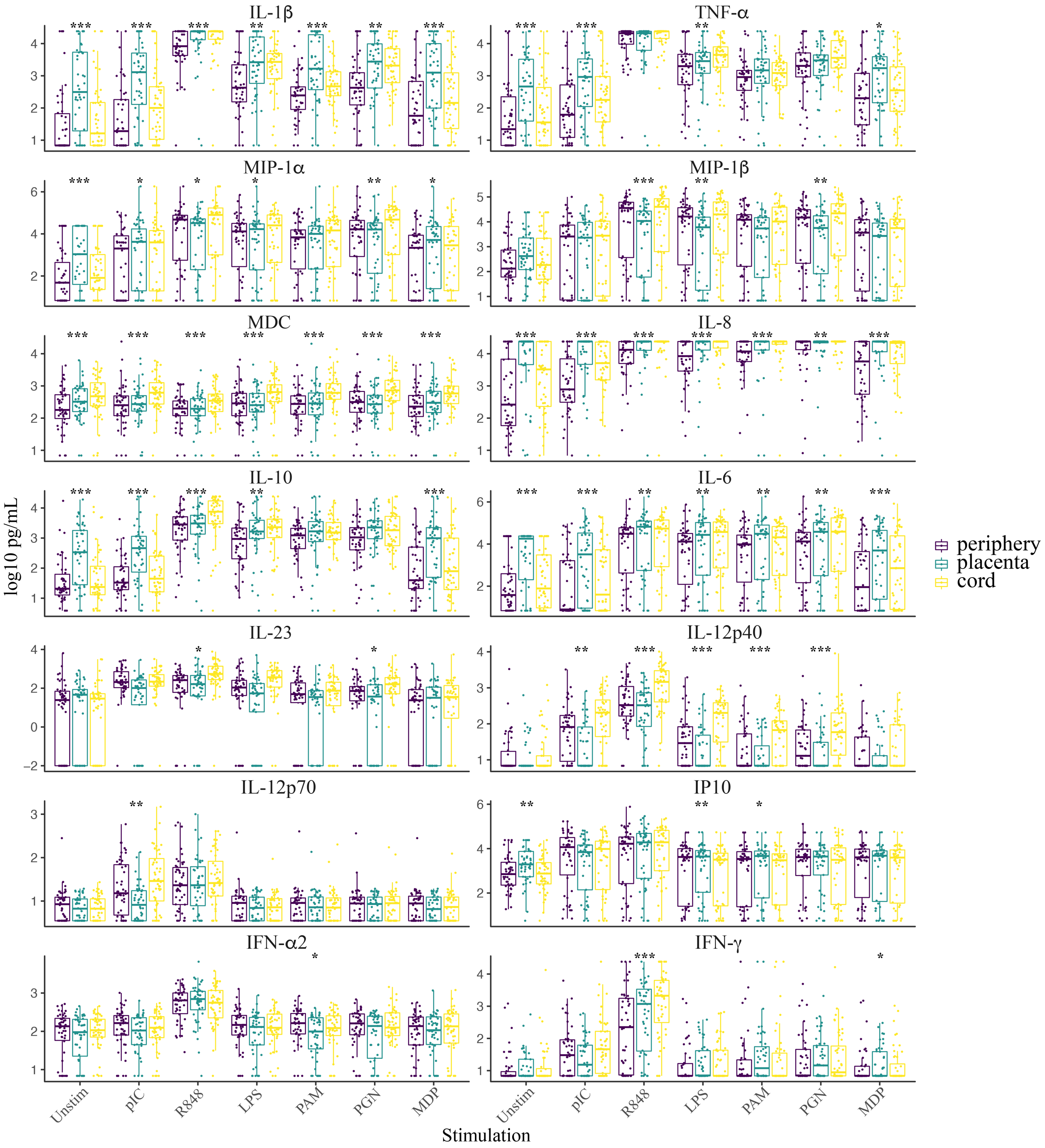

Supplementary Figure 5. Cytokine responses induced innate agonists in peripheral, placental and cord blood. Boxplots showing ratios of cytokines produced upon PRR agonists' stimulations over background unstimulated control. Compartments were compared using the Friedman test (* p-value ≤ 0.05, ** p-value ≤ 0.01; *** p-value ≤ 0.001).

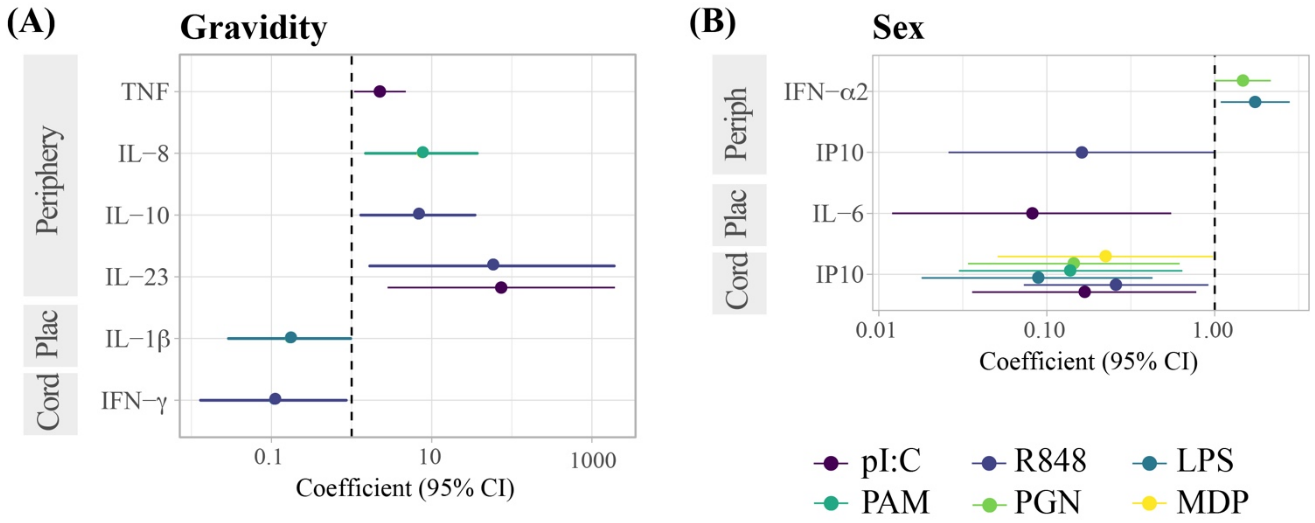

Supplementary Figure 6. Association of gravidity and infant's sex with cytokine responses induced by innate stimulations. Forest plots show the effect of A) unigravidae in reference to multigravidae and of B) male infants in reference to female infants on cytokine responses. Only associations with raw p-values <0.05 are shown (none of the associations were significant after adjusting for multiple testing). The coefficient was calculated as 10^beta and beta was obtained in multivariable regression models adjusted by age (log_{10}-transformed) and technical batch with cytokine ratios (log_{10}-transformed) as outcome.

Table S1. Associations of age with spontaneous cytokine responses.

Compartment	Cytokine	Cytokine group	Coefficient[a]	95% CI	P value[b]	BH P value[c]
Periphery	IL-10	Anti-inflammatory	0.837	0.712 - 0.983	0.031	0.26
Periphery	IL-1β	Pro-inflammatory	0.774	0.617 - 0.97	0.027	0.26
Periphery	IL-8	Pro-inflammatory	0.758	0.592 - 0.97	0.028	0.26
Periphery	MIP-1α	Pro-inflammatory	0.769	0.593 - 0.998	0.049	0.294
Periphery	TNF	Pro-inflammatory	0.794	0.635 - 0.993	0.044	0.294
Periphery	IL-6	TH17	0.709	0.54 - 0.931	0.015	0.26
Placenta	IP10	TH1	1.232	1.041 - 1.457	0.016	0.26

[a] Coefficient show fold-change in the outcome with 10% increases in cytokine concentrations. It was calculated as 1.10^beta and beta was obtained in multivariable regression models adjusted by age (log_{10}-transformed) and technical batch with cytokine concentrations (log_{10}-transformed) as predictor variable and birth outcomes as dependent variable. Birth weight and Ballard score were log_{10}-transformed.

[b] Raw p-value.

[c] Significance was established at Benjamini-Hochberg (BH) adjusted p-value ≤ 0.3. P-values were adjusted for the 42 tests performed.

Table S2. Associations of age with innate cytokine responses to PRR stimulation.

Compartment	Agonist	Receptor	Receptor localization	Cytokine ratio	Cytokine group	Coefficient[a]	95% CI	P value[b]	BH P value[c]
Periphery	R848	TLR7/8	Endosomal	IL-10	Anti-inflammatory	1.243	1.001 - 1.544	0.049	0.95
Periphery	R848	TLR7/8	Endosomal	IL-1β	Pro-inflammatory	1.245	1.007 - 1.54	0.043	0.95
Periphery	PAM	TRL1/2	Surface	IL-1β	Pro-inflammatory	1.168	1.008 - 1.354	0.039	0.95
Periphery	MDP	NOD2	Cytosolic	IL-8	Pro-inflammatory	1.196	1.029 - 1.39	0.021	0.95
Periphery	R848	TLR7/8	Endosomal	IL-8	Pro-inflammatory	1.278	1.026 - 1.592	0.029	0.95
Periphery	PAM	TRL1/2	Surface	IL-8	Pro-inflammatory	1.273	1.033 - 1.567	0.024	0.95
Periphery	R848	TLR7/8	Endosomal	TNF	Pro-inflammatory	1.271	1.014 - 1.594	0.038	0.95
Periphery	LPS	TLR4	Surface	TNF	Pro-inflammatory	1.203	1.002 - 1.445	0.048	0.95
Periphery	PAM	TRL1/2	Surface	TNF	Pro-inflammatory	1.246	1.051 - 1.477	0.012	0.95
Periphery	PGN	NOD1/2	Cytosolic	IL-6	TH17	1.354	1.006 - 1.823	0.046	0.95
Placenta	PAM	TRL1/2	Surface	IL-12p70	TH1	1.051	1.005 - 1.099	0.031	0.95
Placenta	MDP	NOD2	Cytosolic	IP10	TH1	0.785	0.622 - 0.991	0.042	0.95
Cord	PAM	TRL1/2	Surface	IP10	TH1	1.311	1.002 - 1.715	0.048	0.95

[a] Coefficient show fold-change in the outcome with 10% increases in cytokine concentrations. It was calculated as 1.10^beta and beta was obtained in multivariable regression models adjusted by age (log_{10}-transformed) and technical batch with cytokine concentrations (log_{10}-transformed) as predictor variable and birth outcomes as dependent variable. Birth weight and Ballard score were log_{10}-transformed.
[b] Raw p-value.
[c] Significance was established at Benjamini-Hochberg (BH) adjusted p-value ≤ 0.3. P-values were adjusted for all 252 tests performed.

Table S3. Association of spontaneous cytokine production with birth outcomes. Only results with p-values ≤ 0.05 are shown.

Compartment	Cytokine	Cytokine Group	Coefficient (%)[a]	95% CI	P value[b]	BH P-value[c]
**Birth weight**						
Placenta	TNF	Pro-inflammatory	-0.028	-0.052 ; -0.003	0.028	0.588
Placenta	IL-12p40	TH17	-0.073	-0.133 ; -0.013	0.018	0.588
**Fetal hemoglobin**						
Periphery	IL-8	Pro-inflammatory	-0.424	-0.757 ; -0.091	0.014	0.428
Periphery	IL-6	TH17	-0.336	-0.643 ; -0.028	0.033	0.428
**Gestational age**						
Cord	IFN-α2	TH1	-0.022	-0.039 ; -0.005	0.011	0.462

[a] Coefficients show the difference in percentage in the outcome with 10% increases in cytokine concentrations. It was calculated as (1.10^beta-1)*100 and beta was obtained in multivariable regression models adjusted by age (log10-transformed) and technical batch with cytokine concentrations (log_{10}-transformed) as predictor variable and birth outcomes as dependent variable. Birth weight and gestational age were log10-transformed.
[b] Raw p-value. Significance was established at raw p-value ≤ 0.05. A total of 13 tests for each compartment and birth outcome.
[c] Significance was established at a Benjamini-Hochberg (BH) adjusted p-value ≤ 0.3. P-values were adjusted by a total of 42 tests performed for each independent variable.

Table S4. Association of spontaneous cytokine production with prematurity. Only results with p-values ≤ 0.05 are shown.

Compartment	Cytokine	Cytokine group	OR[a]	95% CI	P value[b]	BH P-value[c]
Placenta	MDC	Pro-inflammatory	0.03	0.001 ; 0.331	0.013	0.546
Cord	IL-8	Pro-inflammatory	0.337	0.104 ; 0.915	0.045	0.835

[a] OR: Odds ratio for 10-fold increase in the cytokine concentration. OR were obtained in logistic models adjusted by age (log10-transformed) and technical batch with cytokine concentrations ($\log_{10}$-transformed) as predictor variable and prematurity as outcome.
[b] Raw p-value. Significance was established at raw p-value ≤ 0.05. A total of 13 tests were performed for each compartment.
[c] Significance was established at a Benjamini-Hochberg (BH) adjusted p-value ≤ 0.3. P-values were adjusted by a total of 42 tests performed for each independent variable.

Table S5. Association of cytokines induced by innate stimulations with prematurity. Only results with p-values ≤ 0.05 are shown.

Compartment	Agonist	Receptor	Receptor Localization	Cytokine Ratio	Cytokine	OR[a]	95% CI	P value[b]	BH P-value[c]
**Cord**	MDP	NOD2	Cytosolic	IL-8	Pro-inflammatory	5.339	1.429 ; 28.363	0.022	
**Cord**	PGN	NOD1/2	Cytosolic	IL-8	Pro-inflammatory	3.953	1.381 ; 14.239	0.018	0.841
**Cord**	pI:C	TLR3	Endosomal	IL-8	Pro-inflammatory	4.441	1.113 ; 22.424	0.044	0.841
**Cord**	R848	TLR7/8	Endosomal	IL-8	Pro-inflammatory	3.489	1.193 ; 12.692	0.034	0.841
**Cord**	LPS	TLR4	Surface	IL-8	Pro-inflammatory	4.193	1.386 ; 16.397	0.019	0.841

[a] OR: Odds ratio for 10-fold increase in the cytokine ratio. OR were obtained in logistic models adjusted by age (log10-transformed) and technical batch with cytokine ratios ($\log_{10}$-transformed) as predictor variable and prematurity as outcome.
[b] Raw p-value. Significance was established at raw p-value ≤ 0.05. A total of 78 tests were performed for each compartment.
[c] Significance was established at a Benjamini-Hochberg (BH) adjusted p-value ≤ 0.3. P-values were adjusted by a total of 252 tests performed for each independent variable.
